# Editorial: Analysis of the Interaction of Dp44mT with Human Serum Albumin and Calf Thymus DNA Using Molecular Docking and Spectroscopic Techniques

**DOI:** 10.3390/ijms17111914

**Published:** 2016-11-16

**Authors:** Gregor P. C. Drummen, Christo Z. Christov

**Affiliations:** 1 Cellular Stress and Ageing Program, Hepato and Renal Pathobiology Program, Bio&Nano Solutions, D-33647 Bielefeld, Germany; 2 Department of Applied Sciences, Faculty of Health and Life Sciences, Northumbria University, Newcastle-upon-Tyne NE1 8ST, UK

**Keywords:** Dp44mT, fluorescence quenching, molecular docking, human serum albumin, cytotoxicity, MTT, spectroscopy, circular dichroism

## Abstract

This Editorial refers to:

Merlot, A.M.; Sahni, S.; Lane, D.J.R.; Richardson, V.; Huang, M.L.H.; Kalinowski, D.S.; Richardson, D.R. Letter to the Editor: Analysis of the Interaction of Dp44mT with Human Serum Albumin and Calf Thymus DNA Using Molecular Docking and Spectroscopic Techniques. *Int. J. Mol. Sci*. **2016**, *17*, 1916.

Xu, Z.; Liu, Y.; Zhou, S.; Fu, Y.; Li, C. Response to Letter to the Editor by D. Richardson: Analysis of the Interaction of Dp44mT with Human Serum Albumin and Calf Thymus DNA Using Molecular Docking and Spectroscopic Techniques. *Int. J. Mol. Sci*. **2016**, *17*, 1917.

Xu, Z.; Liu, Y.; Zhou, S.; Fu, Y.; Li, C. Correction: Analysis of the interaction of Dp44mT with human serum albumin and calf thymus DNA using molecular docking and spectroscopic techniques. *Int. J. Mol. Sci*. **2016**, *17*, 1915.

Xu, Z.; Liu, Y.; Zhou, S.; Fu, Y.; Li, C. Analysis of the Interaction of Dp44mT with Human Serum Albumin and Calf Thymus DNA Using Molecular Docking and Spectroscopic Techniques. *Int. J. Mol. Sci*. **2016**, *17*, 1042.

In a recent *Internal Journal of the Molecular Sciences* (*IJMS*) publication, Xu and co-workers presented evidence on the pharmacological interaction of the anti-tumor agent di-2-pyridylketone-4,4-dimethyl-3-thiosemicarbazone (Dp44mT) with human serum albumin (HSA) and DNA [1]. The pharmacological interaction of Dp44mT is an important issue in the optimization of Dp44mT as an anti-tumor agent [2]. To unravel the aforementioned interaction, the authors used a combination of fluorescence, UV-VIS absorbance, and circular dichroism measurements and molecular docking techniques.

However, upon publication, this paper became the focus of controversy and was alleged to contain numerous important flaws and factually incorrect statements [3]. Furthermore, the performed measurements were perceived as non-optimal and potentially problematic, and thus, the results and their interpretation might be unreliable. Because *IJMS* takes any complaint that is brought before us serious, *IJMS* started an investigation and asked Xu et al. to reply to the letter in detail [4] and assigned independent experts to reevaluate the manuscript and the correspondence regarding this manuscript. In addition, we, as members of the editorial board, evaluated the issues brought before us.

The overall consensus was that the alleged controversial nature of the paper was not significant enough to warrant a retraction or extensive correction. Xu et al. could certainly have used additional methods or more control experiments, but this is an issue that is not unique for this paper. We feel that the paper by Xu et al. provides new insights into the pharmacological action of Dp44mT and fits well within a climate that allows and stimulates scientific discussion. Nonetheless, two issues raised in the letter to the editor [3] were identified that required correction/amendment, which are published in a corrigendum [5].

Finally, *IJMS*, its Editorial Board, the handling editors and MDPI uphold the highest publication standards. *IJMS* is a member of the Committee on Publication Ethics (COPE). We fully adhere to its Code of Conduct and to its Best Practice Guidelines (see also: http://www.mdpi.com/journal/ijms/instructions). The current thorough investigation in which the opinion of external experts was solicited exactly demonstrates the adherence to such high standards.

## Abbreviations

MDPI	Multidisciplinary Digital Publishing Institute
IJMS	International Journal of Molecular Sciences
COPE	Committee on Publication Ethics
